# Neuronal Activity in the Subthalamic Cerebrovasodilator Area under Partial-Gravity Conditions in Rats

**DOI:** 10.3390/life4010107

**Published:** 2014-03-04

**Authors:** Jorge L. Zeredo, Kazuo Toda, Yasuhiro Kumei

**Affiliations:** 1 Graduate School, Tokyo Medical and Dental University, Tokyo 113-8549, Japan; 2 University of Brasilia, Brasilia 72220-140, DF, Brazil; E-mail: jllzeredo@unb.br; 3 Graduate School, Nagasaki University, 1-7-1 Sakamoto, Nagasaki 852-8588, Japan; E-Mail: k-toda@nagasaki-u.ac.jp

**Keywords:** parabolic flight, gravity, threshold, electrophysiology, brain, subthalamic cerebrovasodilator area, cerebral blood flow, partial gravity, intracranial EEG, rats

## Abstract

The reduced-gravity environment in space is known to cause an upward shift in body fluids and thus require cardiovascular adaptations in astronauts. In this study, we recorded in rats the neuronal activity in the subthalamic cerebrovasodilator area (SVA), a key area that controls cerebral blood flow (CBF), in response to partial gravity. “Partial gravity” is the term that defines the reduced-gravity levels between 1 g (the unit gravity acceleration on Earth) and 0 g (complete weightlessness in space). Neuronal activity was recorded telemetrically through chronically implanted microelectrodes in freely moving rats. Graded levels of partial gravity from 0.4 g to 0.01 g were generated by customized parabolic-flight maneuvers. Electrophysiological signals in each partial-gravity phase were compared to those of the preceding 1 g level-flight. As a result, SVA neuronal activity was significantly inhibited by the partial-gravity levels of 0.15 g and lower, but not by 0.2 g and higher. Gravity levels between 0.2–0.15 g could represent a critical threshold for the inhibition of neurons in the rat SVA. The lunar gravity (0.16 g) might thus trigger neurogenic mechanisms of CBF control. This is the first study to examine brain electrophysiology with partial gravity as an experimental parameter.

## 1. Introduction

Most astronauts experience an array of uncomfortable symptoms during acclimation to microgravity [1]. These include headache, vomiting, nausea, lethargy, and gastric discomfort, which typically last 2 to 3 days [2] but may sometimes last considerably longer [1]. At this point it is important to clarify the causes of these symptoms because they may significantly impair the performance capacity of astronauts, and because the current treatments involve drugs that have undesirable side-effects [2]. Spaceflight has effects on blood and other body fluids dynamics and control [3,4]. It also can induce intracranial hypertension and alter brain activity and can influence the occurrence of headaches and other symptoms [5,6,7]. Recently, in the rat, a limited area of the subthalamus has been implicated in controlling cerebral blood flow (CBF) [8]. Neurons in this area, termed the subthalamic cerebrovasodilator area (SVA), were shown to relay sensory signals in order to regulate CBF to the forebrain [8]. In this study, we hypothesized that acute exposure to low gravity would modulate the activity of neurons in the SVA of the rat. To test this hypothesis, neuronal activity in the SVA was recorded telemetrically through chronically implanted microelectrodes in freely moving rats during exposure to partial gravity (*i.e*., the reduced-gravity levels between the 1 g of Earth and 0 g of space).

## 2. Experimental Section

### 2.1. Animals Selection and Preparation

A total of 16 male Wistar albino rats weighing 230 to 250 grams (SLC, Shizuoka, Japan) were used in this experiment. Care and use of rats compiled with the guidelines for animal welfare in Japan and received institutional approval by the Animal Welfare Committees of Tokyo Medical and Dental University (No. 80079A), Japanese Space Agency (No. 007-026), and Nagasaki University (No. 0507050445). Rats were individually housed and kept at the temperature of 20–23 °C on a 12 h-each light/dark cycle, and had free access to food and water in the laboratory.

Neuronal activity in the SVA was recorded by chronically implanted electrodes (one in each rat). Brain electrodes were surgically implanted at Nagasaki University, division of Integrative Sensory Physiology, by one of the authors (J.Z.). Each rat was deeply anesthetized with intraperitoneal thiamylal sodium (80 mg/kg) and placed on a stereotaxic frame (Narishige Co., Tokyo, Japan). First, the hair on the head was trimmed with animal clippers and the skin was disinfected with iodine povidone (Isojin, Meiji Seika, Japan). The scalp was incised and the skin and periosteum were deflected. Then, following stereotaxic coordinates and anatomical landmarks on the bone, a small hole was drilled on the skull with a round #2 stainless steel bur for electrode implantation. A single tungsten needle-electrode (9–12 MΩ impedance; FHC, Bowdoin, ME, USA) was lowered vertically into the SVA following stereotaxic coordinates [9], −4.5 mm caudal from bregma, 1.2 mm lateral from the midline, and −7.0 mm ventral from the surface of the brain according to the stereotaxic coordinates. Electrode insertion was guided by a micromanipulator (SM-15, Narishige, Tokyo, Japan). The electrode was fixed to the skull by stainless steel screws and cyanoacrylate glue (Toa-Gosei Co. Ltd., Tokyo, Japan). A ground wire was fixed to one of the screws. The electrode and ground wire were attached to a miniature electronic connector, and the exposed attachments were isolated with epoxy glue (Loctite, Henkel, Rocky Hill, CT, USA). The connector was then fixed to the skull with cyanoacrylate glue and acrylic resin (Unifast II, GC, Tokyo, Japan). After setting of the acrylic, the skin flap was returned and the wound was sutured (Vicryl, Ethicon, Somerville, NJ, USA) leaving only the electronic connector slightly protruding out of the skin. Finally, the animals were allowed to recover from anesthesia on a warm electric blanket and then returned to their cages.

### 2.2. Parabolic Flight

**Figure 1:**
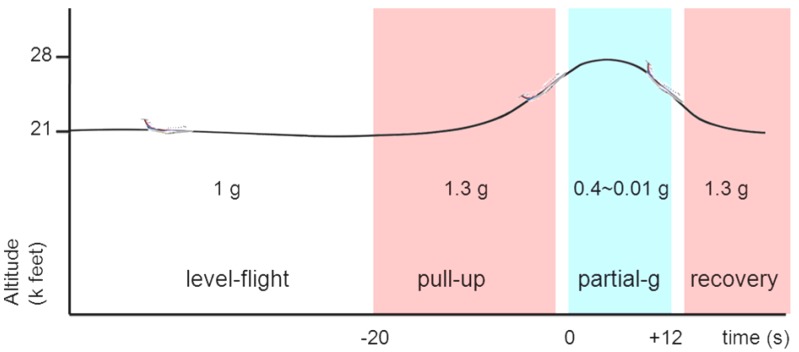
Scheme of parabolic maneuver. The pull-up phase of the parabola was set to generate a mild hypergravity of 1.3 g in order to minimize the any possible physiological response to hypergravity exposure in the animals.

Two to four days after surgery (the animals were operated over the period of three days), the rats were transported to a laboratory at Nagoya Airport where the rats were acclimated for another three to five days in order to minimize the stress of the transport and relocation. Parabolic flights were operated by Diamond Air Service, Inc. (Toyoyama-cho, Japan) on a Mitsubishi MU300 jet aircraft. The rats were accommodated individually in flight chambers (dimensions: 40 × 40 × 40 cm) that were fixed to the flight deck by a metal frame and surrounded completely by thick black curtains. The flights had a total approximate duration of 2 h, including take-off, transit to and from the test area, and landing. We customized an original parabolic-flight trajectory as reported previously [10]. After 1 g level-flight, the aircraft entered into the “pull-up” phase of approximately 20 s at 1.3 g hypergravity, then diving into the target partial-gravity that lasted 10–15 s, followed by the “recovery” phase of 20 s at 1.3 g (Figure 1). Our parabola trajectory uses 1.3 g for the hypergravity phases of “pull-up” and “recovery”, which is different from the conventional parabolic flights that use 1.8–2 g The 2 hr flight included 12–14 parabolas, each of which with a specific target partial-gravity level. In each flight, a set of four different partial-gravity levels was repeated three times on the same flight. Neither food nor water was supplied during the flight. The temperature inside the cabin was kept at 20–23 °C.

### 2.3. Data Collection

Data was recorded through a telemetry system (Dia Medical, Tokyo, Japan). Immediately before the flight, the rats were manually restrained and worn a jacket (Harvard Apparatus, Holliston, MA, USA) containing a miniature FM transmitter and a button cell battery (Dia Medical, Tokyo, Japan). The transmitter was connected to the electrode on the rat’s head via a short cable. The transmitter was disc-shaped with 15 mm in diameter and 2 mm in thickness, and weighed 1 gram. Signals of spontaneously active units in the rat SVA were recorded and transmitted to antennas placed just outside the test box and then to a receiver/amplifier (10× differential amplification; DTT-1000, Dia Medical, Tokyo, Japan). The signal was then digitalized (Micro1401, Cambridge Electronic Design Ltd., Cambridge, UK), with the sampling rate of 10 kHz, and stored in a laptop computer running the Spike2 software (Cambridge Electronic Design Ltd., Cambridge, UK). Waveforms were sampled throughout the entire flight. Unit spikes were identified from waveforms recorded between 0.3 and 3 kHz and isolated by template matching (Spike2, Cambridge Electronic Design Ltd., Cambridge, UK). This technique allows us to identify the activity of individual neurons and to distinguish between neuronal activity and background noise [11]. Single units were isolated from each rat’s electrophysiological recordings with the Spike2 software. A total of 140 samples were used in the analysis.

### 2.4. Data Analysis

The firing frequency in each phase of the parabola trajectory was calculated as the total number of spikes divided by the length (seconds) of the respective phase. The firing frequency in the low-gravity phase was compared with that in the preceding 1 g level-flight and 1.3 g “pull-up” phases. The effect of different g-levels on neuronal firing frequency was analyzed by one-way repeated-measures ANOVA, and in case statistically significant difference was found, paired t-tests were used to compare the firing frequency at low-gravity and at the immediately preceding 1 g phase. A two-way ANOVA was conducted to examine habituation effect of repeated exposures on the SVA firing frequency.

### 2.5. Histology and Localization of Electrode Insertion Site

In order to localize the electrode’s insertion site histologically, after the flight experiment we deeply anesthetized the rats and passed a weak anodal current (0.3 mA, 20 s) through the recording electrodes. This step produces an electrolytic mark that helps to identify the position of the electrode’s tip on a histological section. Then, the rats were decapitated and had their brains removed, fixated in 10% paraformaldehyde solution, and embedded in paraffin wax. Serial sections (8–15 µm) in the coronal plane were prepared and stained with either Hematoxylin-Eosin or Nissl. The electrode position was estimated as the center of the electrolytic mark as superimposed in the rat’s brain Atlas [9].

## 3. Results and Discussion

**Figure 2:**
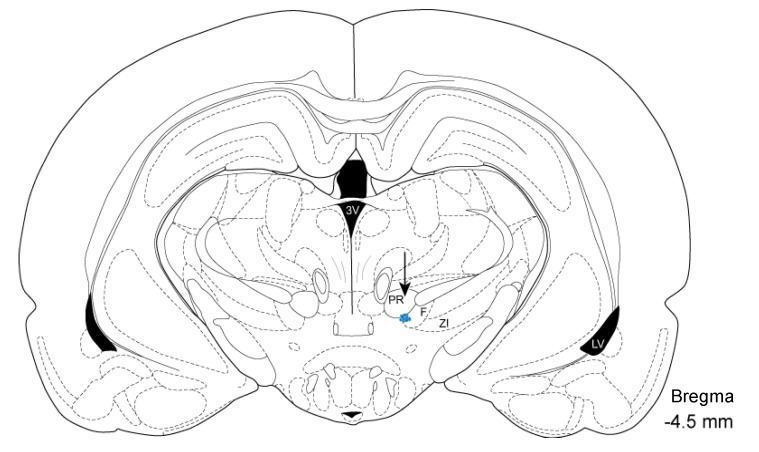
Schematic drawing of the electrodes position. Histological sections each of the brains were superimposed with the corresponding drawing from the Paxinos Atlas, confirming the electrode position within the subthalamic cerebrovasodilator area (SVA). Arrow indicates blue dots representing the center of electrolytic lesions by an anodal current (0.3 mA, 20 s) passed through the recording electrode. PR: prerubral field, F: fields of Forel, ZI: zona incerta, 3V: thirdventricle, LV: lateral ventricle.

**Figure 3:**
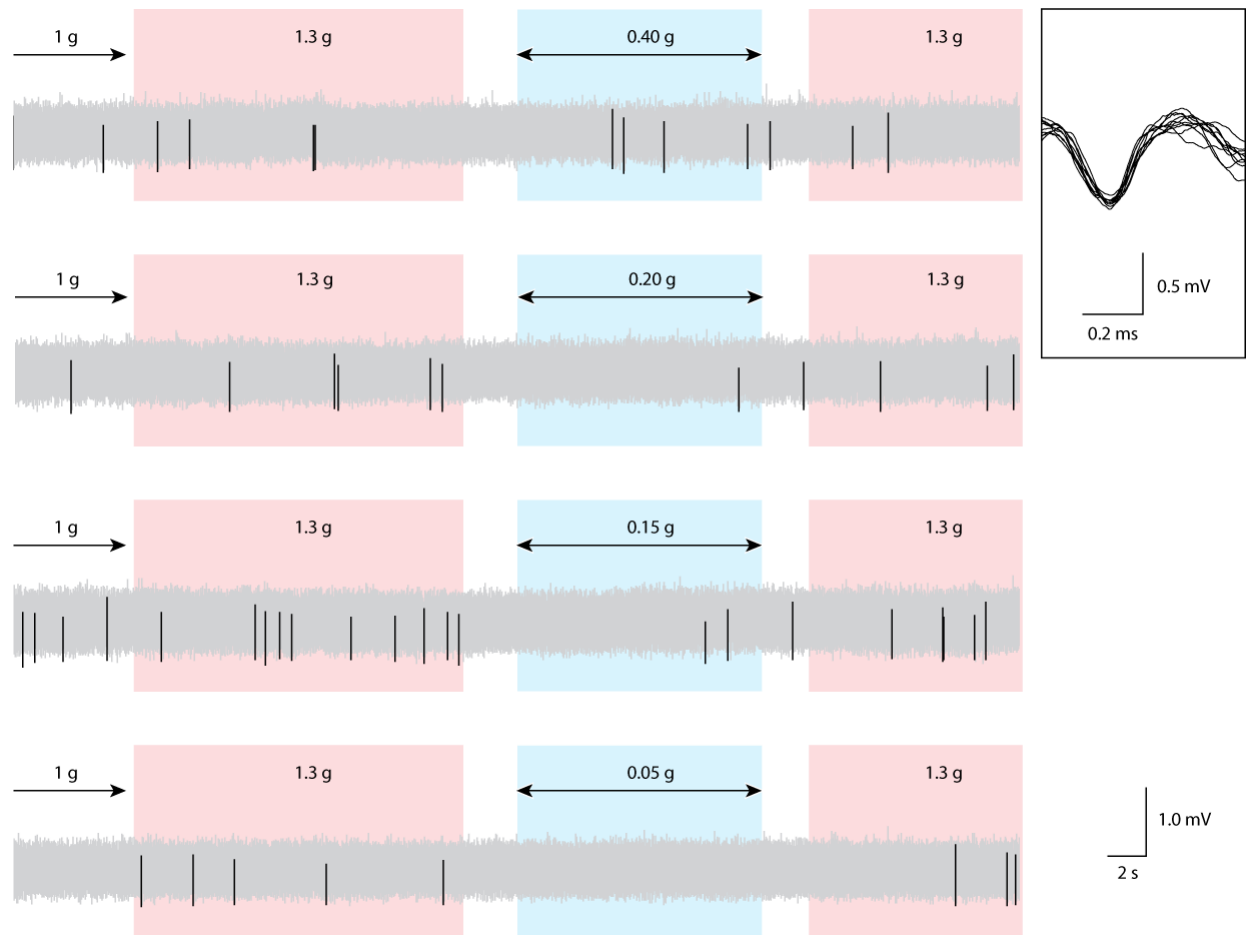
Neuronal activity in the rat SVA during parabolic flight. Sample waveforms are shown in light-gray with template-matched unit spikes shown in black. From the top, samples from the same animal at partial-gravity levels 0.40, 0.20, 0.15, and 0.05 g are shown. Top inset tracing shows 10 superimposed consecutive spikes from the targeted template. The parabolic flight was composed of stable 1 g straight-and-level flight (baseline data), 1.3g pull-up phase, partial-gravity phase (variable), and 1.3g recovery phase.

Electrophysiological recordings were obtained from 16 male Wistar albino rats. Histological examinations confirmed the electrode position within the SVA (Figure 2). Compared to 1 g level-flight, the average single-unit firing frequencies were lower during exposures to gravity levels of 0.15, 0.1, 0.05, and 0.01 g. Spike histograms showed changes in SVA activity in relation to the different phases of the parabolic flight, including 1 g level-flight, partial gravity, and the phases of “pull-up” and “recovery” at 1.3 ± 0.1 g (Figure 3).

Partial-gravity stimulation from 0.4 g through 0.2 g did not significantly affect the firing rate of the unit activity in rat SVA. On the other hand, all the stimulations by partial gravities from 0.15 g through 0.01 g decreased the firing rate significantly, as compared to each precedent 1 g level-flight (Table 1). All the rats showed spontaneous spiking in the SVA with the average firing frequency of 2.30 ± 1.68 Hz (mean ± SD, n = 140) during the 1 g level-flight. The firing frequency during “pull-up” phase was not significantly different from the corresponding precedent 1 g level-flight (*p *= 0.25). A decrease in the average firing frequency at the partial gravity phase was observed in comparison to the preceding 1 g level-flight. Repeated-measures ANOVA showed that the average firing frequency was significantly different in relation to g-level stimulation (*F* (1,6) = 21.905, *p *= 0.016). Paired t-tests showed that the average firing frequencies were not significantly different during 0.4 g, 0.3 g, and 0.2 g in comparison to the preceding 1 g level-flight, but significantly lower during 0.15 g (average difference = −1.05 ± 1.65 Hz, n = 20, *p* = 0.010), 0.1 g (−0.53 ± 1.18 Hz, n = 37, *p* = 0.009), 0.05 g (−0.79 ± 1.13 Hz, n = 29, *p* = 0.001), and 0.01 g (−1.16 ± 1.29 Hz, n = 9, *p *= 0.027).

We also examined the habituation effects of repeated exposures to partial gravity on neuronal activity in the SVA. In the two-way ANOVA, the effect of number of exposures and gravity level on neuronal response in the rat SVA showed no significant interaction, *F* (6,12) = 0.885, *p* = 0.564. (But see “Limitations”, below.)

We recently reported the occurrence of stereotypical behaviors in relation to the level of low-gravity exposure in the rat [10]. Therefore, to the best of our knowledge, this is the second report to use gravity levels between 1 g and 0.01 g (termed partial gravities) as the experimental parameter with brain electrophysiology as the experimental end-point. The original flight trajectory used here reduced the magnitude hypergravity phase of the parabola from the usual 2 g to 1.3 g; thus minimizing the effects of hypergravity on the observed responses to low gravities.

In this study, we tested several levels of low-gravity stimulation on the electrophysiological responses of neurons the SVA, which is believed to be a key area in neuronal circuits that controls regional blood flow within the brain [8]. Our results show that exposure to gravity levels lower than 0.2 g had the effect of decreasing the electrical activity in neurons of the SVA.

**Table 1:** Neuronal activity in the rat SVA at different partial-gravity levels.

Partial gravity (g)	Number of recorded samples	Number of rats	Firing frequency during 1 g (Hz)	Firing frequency at each partial g (Hz)	Average difference (Hz)	Value of *p*
0.40	9	3	1.57 ± 0.64	1.56 ± 0.75	0.00 ± 0.76	0.981
0.30	11	3	1.31 ± 0.53	1.36 ± 0.73	0.30 ± 1.32	0.798
0.20	25	8	1.86 ± 0.88	1.47 ± 0.96	−0.39 ± 1.21	0.078
0.15	20	8	3.14 ± 2.56	2.09 ± 1.23	−1.05 ± 1.65	0.001 **
0.10	37	12	2.15 ± 1.49	1.62 ± 0.88	−0.53 ± 1.18	0.003 **
0.05	29	12	2.46 ± 1.69	1.67 ± 0.97	−0.79 ± 1.13	0.001 **
0.01	9	3	3.68 ± 1.96	2.52 ± 0.98	−1.16 ± 1.29	0.013 *

Repeated measures ANOVA showed significant difference in the effect of partial-gravity on the mean firing rate (*p* = 0.016). Statistical significance in Fisher’s PLSD post-hoc: * *p* < 0.05 ** *p* < 0.01; Data are expressed as the mean ± standard deviation.

Unlike most tissues in the body, blood flow to the brain is always maintained at a relatively constant level. This is true even during strenuous exercise, when most of the increased cardiac output is directed towards the skeletal muscles, whereas blood flow to the kidneys and digestive organs declines. The redistribution of blood during exercise is mediated by mechanisms that include myogenic, neurogenic and endocrine controls. In space, one of the most prominent cardiovascular phenomena is a redistribution of body fluids toward the head [3,4]. In head-down tilt experiments, redistribution of body fluid similar to that observed in space has been reported to raise the intracranial pressure in humans [4] and in the rabbit [12]. However, parabolic-flight experiments have shown that CBF returns to normal within 3 seconds of low-gravity (0.04 g) exposure in rats [13]. In such short-term, the increase in intracranial pressure is compensated by myogenic and neurogenic controls, which regulate the diameter of cerebral vessels to keep CBF constant [14]. Myogenic controls occur when the stretch of arterial walls directly increases the tone of vascular smooth muscles, thus causing constriction of cerebral vessels in response to increased blood pressure. Neurogenic controls rely on vasomotor (autonomic) reflexes originating from mechanoreceptors and chemoreceptors located on the walls of cerebral vessels [15]. Previous studies indicate that neurogenic controls play a role in fine-tuning CBF during head-down tail suspension (an experimental situation that simulates the accumulation of blood at the upper body extremities, as in microgravity) [16,17].

Electrical stimulation of the SVA in rats was shown to increase CBF in a frequency and current dependent fashion [8]. In natural circumstances, the SVA is excited by sympathoexcitatory neurons of the rostral ventrolateral medullary nucleus (RVLM) [8], which elevates CBF [18,19,20]. Conversely, a decrease in the electrical activity in this area would be consistent with a decrease in CBF. Indeed, studies have shown that exposure to microgravity (during parabolic flights) induced a reduction in sympathetic tone in response to the body-fluid shift in humans [21,22] and in the rat [23]. Therefore, it is conceivable that a neurogenic vasoconstriction mediated by SVA neurons could help to counteract the sudden increase in intracranial pressure caused by low-gravity exposure.

### Limitations

There are a number of potential limitations to this study. First and foremost, it is difficult to maintain stable neuronal recordings over long periods of time, especially considering the demands inherent to a p-flight experiment, such as strict operational schedule and unexpected/uncontrollable vibrations. Therefore, we cannot guarantee that the recording conditions were the same throughout the experiment. This should be taken into consideration when we compare time-distant events, such as the effect of repeated exposures on neuronal activity. All other comparisons that we describe here were made between a low-gravity phase and its immediately preceding 1 g level-flight or 1.3 g “pull-up” phases of the flight. In addition, different numbers of samples were obtained from each partial-gravity level, which possibly influenced the results of the statistical analysis. Another limitation was that the temporal resolution of the gravity-stimulation profile did not allow for the detection of any instantaneous (or “phasic”) neuronal response; therefore, in this study we limited the analysis to tonic responses by means of average firing frequency comparisons. 

## 4. Conclusions

The level of low-gravity between 0.2–0.15 g could represent a critical threshold for triggering neurogenic controls of blood flow within the brain through activity in the SVA. Modulation of neuronal activity in the SVA may be one of immediate early responses to lower gravities in rats. Failure in such homeostatic mechanism may be at least in part responsible for maladaptive responses in low gravity. If the same mechanism is verified in humans, knowledge brought by this study may help to better design countermeasures against partial- and microgravity environments.
